# Factors Underlying Failure of Methotrexate Treatment in Rheumatoid Arthritis: Implications in Personalized Care

**DOI:** 10.26502/aimr.0203

**Published:** 2025-04-22

**Authors:** Ananta Srivastava, Stefanie Au, Sumanjali Reddy Kanmantha Reddy, Emmanuel Katsaros, Devendra K. Agrawal

**Affiliations:** 1 Departments of Translational Research, College of Osteopathic Medicine of the Pacific, Western University of Health Sciences, Pomona, California 91766 USA; 2 Internal Medicine, College of Osteopathic Medicine of the Pacific, Western University of Health Sciences, Pomona, California 91766 USA

**Keywords:** Autoantibodies, Autoimmune disease, B-cell depletion therapy, Corticosteroid, Disease-modifying antirheumatic drugs, Genetic polymorphism, IL-6 antagonist, Inflammation, JAK inhibitor, Methotrexate, Methotrexate metabolism, NSAIDs, Rheumatoid arthritis, SNPs, TNF inhibitor

## Abstract

Rheumatoid arthritis (RA) is a chronic inflammatory disease that can be managed with a range of therapeutic treatments. Methotrexate (MTX) is a first-line treatment for RA; however, its metabolism in RA patients can be complicated by multiple factors. Therefore, understanding these specific factors is crucial for optimizing the efficacy of MTX to provide improved therapeutic outcomes for patients. This article explores existing literature to examine how MTX metabolism in RA patients is impacted by other commonly used medications for RA. Additionally, the review explores the role of genetics by investigating the impact that single nucleotide polymorphisms (SNPs) have on MTX metabolism. Key findings from this review highlight how MTX metabolism can be enhanced or impaired based on specific combination therapies and how alternative treatments are considered with MTX treatment failure. MTX metabolism can also vary across different racial, ethnic, and population-based groups due to the presence of distinct SNPs in their genetic profiles. These results underscore the importance of personalized treatment approaches when treating RA patients with MTX, as its metabolism is influenced by factors such as drug interactions and SNPs. Future research is needed to expand our understanding of these factors to further improve therapeutic outcomes in RA patients.

## Introduction

Rheumatoid arthritis (RA) is a chronic autoimmune disease characterized by the immune system attacking the lining of the joint [1,2]. The hallmark symptoms of RA include symmetrical joint pain, particularly in the hands and feet, along with joint stiffness in the morning that typically lasts more than 30 minutes [1,3]. RA may manifest in other organs, such as the lungs, heart, and eyes while presenting with pericarditis, pleural effusion, or interstitial lung disease [1,3]. As there is no single diagnostic method that is considered the gold standard for RA, its diagnosis relies on clinical evaluation and is supported by the presentation of symptoms, serologic tests, and imaging findings [1,3,4]. Additionally, key laboratory tests for RA include rheumatoid factor and anti-cyclic citrullinated antibodies [1,3,5,6]. However, despite the presence of disease, one in three individuals may still test negative for these markers (seronegative) [3].

Certain risk factors can increase the likelihood of developing RA. Some examples include smoking, obesity, a family history of the disease, and genetics [3,4]. RA is also debilitating, with 80% of patients who are not properly treated having misaligned joints and 40% being unable to continue working within a decade from the manifestation of the disease [7]. Therefore, early and effective treatment is crucial. While there have been advances in the diagnosis and management of RA, challenges still exist with achieving an early diagnosis and timely referral of patients to the appropriate specialists [4].

One of the primary treatments used for RA is methotrexate (MTX), a disease-modifying antirheumatic drug (DMARD). MTX alters the activity of dihydrofolate reductase, a part of the folate metabolism pathway, resulting in the inhibition of DNA synthesis and purine metabolism [7,8]. It is usually administered as a weekly dose and is often given as an initial monotherapy for patients with moderate to severe RA [1,3]. MTX is not fully effective as a treatment, as only an estimated 40%–50% of RA patients achieve disease remission or have reduction in disease activity [1,9]. While the medication possesses potent anti-inflammatory and anti-proliferative properties, the individual treatment responses can differ due to a myriad of underlying factors [10].

One retrospective study in a tertiary care hospital in Pakistan identified that RA patients who had an increased body mass index (BMI), diabetes, being female, and smoking correlated with increased risk of MTX treatment failure [11]. Another crucial factor that can influence responsiveness to MTX in individuals is genetic polymorphisms [10]. Identifying variations in gene polymorphisms can aid as a tool for understanding the effectiveness and progression of MTX treatment in RA patients [10,12]. Previous studies have identified 120 single nucleotide polymorphisms (SNPs), a type of genetic polymorphism, that may potentially impact MTX treatment response in RA patients [13]. The review critically examined the current literature on the existing therapeutic options and their connections to treatment with MTX for RA patients, SNPs that influence MTX metabolism in these patients, and explored potential variations in gene polymorphisms across different racial, ethnic, and population-based groups.

## Method

To select research findings for this article on MTX metabolism in RA patients, with a focus on current therapeutics, SNPs, and potential polymorphisms prevalent in different groups, a comprehensive search was conducted using databases such as PubMed and Google Scholar from 2005 – 2025. Key search terms included: “MTX metabolism,” “rheumatoid arthritis,” “SNP,” “DMARD,” “corticosteroid,” “conventional synthetic DMARD,” “TNF inhibitor,” “IL-6 antagonist,” “B-cell depletion therapy,” “JAK inhibitors,” and “NSAID.” Articles excluded from consideration were those not written in English or those available only as abstracts.

### Prevalence and Incidence

**Figure 1:**
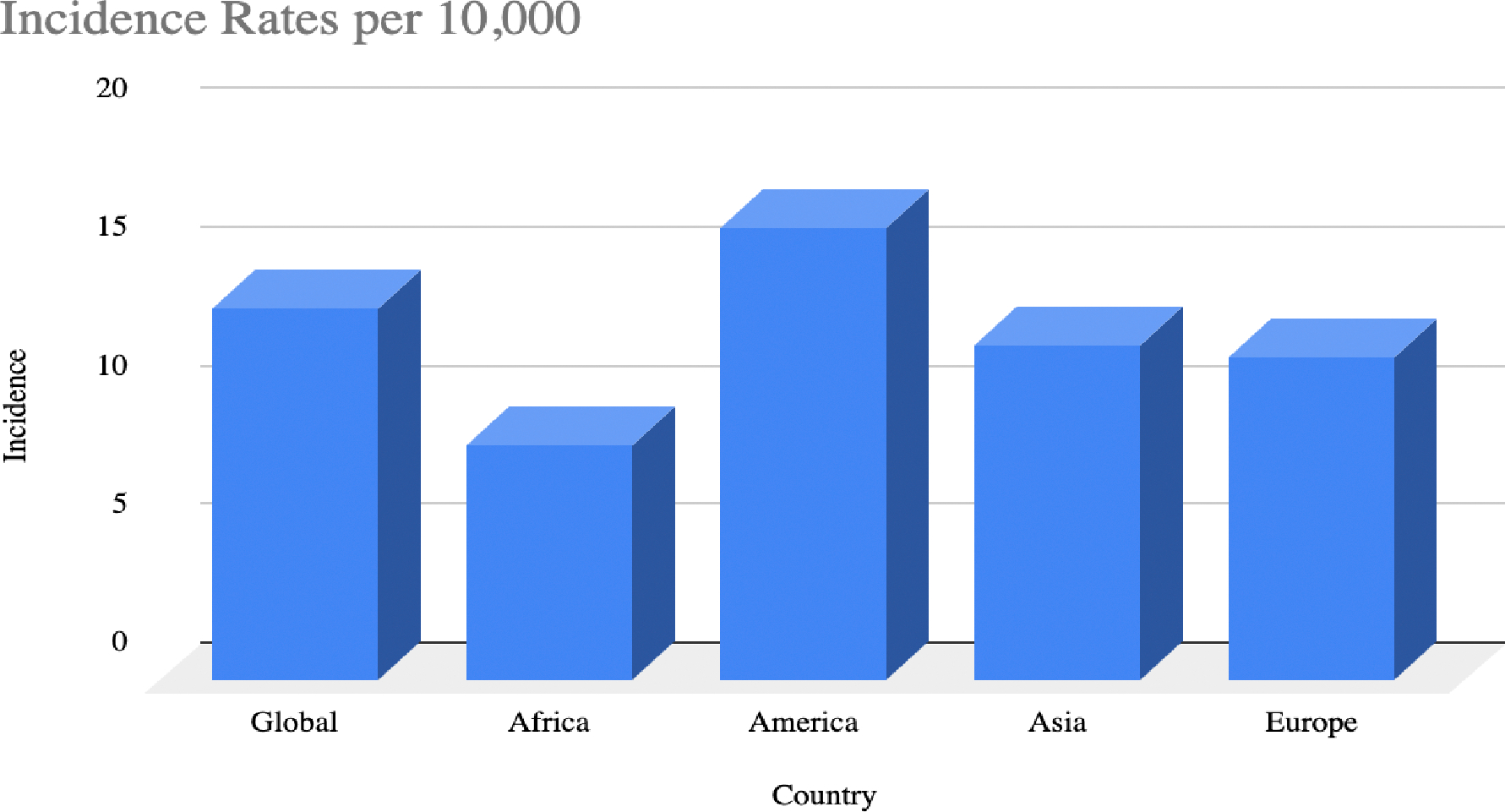
Incidence Rates of RA. This table demonstrates the global incidence of rheumatoid arthritis and a breakdown of the rates per 10,000 in different continents.

The global prevalence of RA varies, with higher rates reported in industrialized countries, likely influenced by environmental exposures, genetic predispositions, and demographic differences [14,71] (Figure 1). In the United States (U.S.), RA affects approximately 1.3 million adults, representing 0.6% to 1% of the population [15,16]. As previously mentioned, common risk factors include both non-modifiable factors, such as genetics and sex, and modifiable factors, such as smoking and obesity [3,4,15]. The disease imposes significant societal and individual burdens, with approximately 35% of patients experiencing work disability. Indirect costs, driven by lost work productivity, are estimated to be nearly three times greater than direct treatment costs. In the U.S. alone, the total annual healthcare expenditure for RA is approximately $19.3 billion [15,17].

The prevalence of RA may have increased in recent years, with emerging evidence suggesting that infections, particularly COVID-19, may influence the incidence and exacerbation of autoimmune diseases like RA. A large population-based study conducted from 2018 to 2023 found that individuals who developed COVID-19 had twice the incidence rate of RA-related diagnoses compared to those without COVID-19. This aligns with earlier research highlighting infections as environmental triggers for autoimmune processes. While these findings emphasize a potential link, further longitudinal studies are needed to explore contributing factors, such as the effects of vaccination, other environmental exposures, and long-term outcomes in RA development after COVID-19 [18].

### Pathophysiology of Rheumatoid Arthritis

The autoimmune response in RA is driven by several inflammatory pathways, with Nuclear Factor Kappa-Β (NF-κB) playing a central role in regulating transcription factors that amplify inflammation. Activated by pro-inflammatory cytokines such as IL-1, TNFα, and IL-6, NF-κB promotes joint inflammation, bone erosion, and disease progression. Another critical pathway involves the Receptor Activator of Nuclear Factor Kappa-Β Ligand (RANKL), which drives osteoclast differentiation and bone destruction, contributing to the osteoclastogenesis and bone degradation by activated NF-κB [19]. The persistent production of these cytokines disrupts the balance of bone remodeling, favoring resorption and leading to progressive joint damage and systemic bone loss.

Joint swelling in RA results from synovial inflammation driven by immune activation and leukocyte infiltration into the synovial compartment. This complex interplay involves both innate and adaptive immune responses, with dendritic cells (DCs), monocytes, mast cells, innate lymphoid cells, T-helper cells, B cells, and plasma cells playing significant roles. Environmental and genetic factors can provoke DCs, which activate T cells and stimulate B cells, macrophages, synoviocytes, and osteoclasts. These cells release inflammatory mediators such as IL-1β, IL-6, TNF-α, and matrix metalloproteinases (MMPs), perpetuating chronic inflammation and driving tissue remodeling. The integration of these immune pathways in the synovium and adjacent bone marrow perpetuates inflammation, tissue damage, and leukocyte migration into affected joints [20].

The synovial tissue undergoes profound structural changes that contribute to joint destruction in RA. The hyperplastic synovial lining layer, composed of synovial fibroblasts (SFs) and macrophages, forms an invasive pannus that erodes cartilage and subchondral bone. The sub-lining layer, infiltrated with immune cells and enriched with memory CD45RO+ T cells, further supports inflammation. These T cells, drawn by chemokines and sustained by survival signals like IL-7 and IL-15, create a persistent inflammatory environment. SFs in RA adopt an aggressive, tumor-like phenotype, acting similarly to T and B cells in producing matrix-degrading enzymes, such as MMPs and cathepsins, that degrade cartilage and secrete RANKL [21].

**Figure 2:**
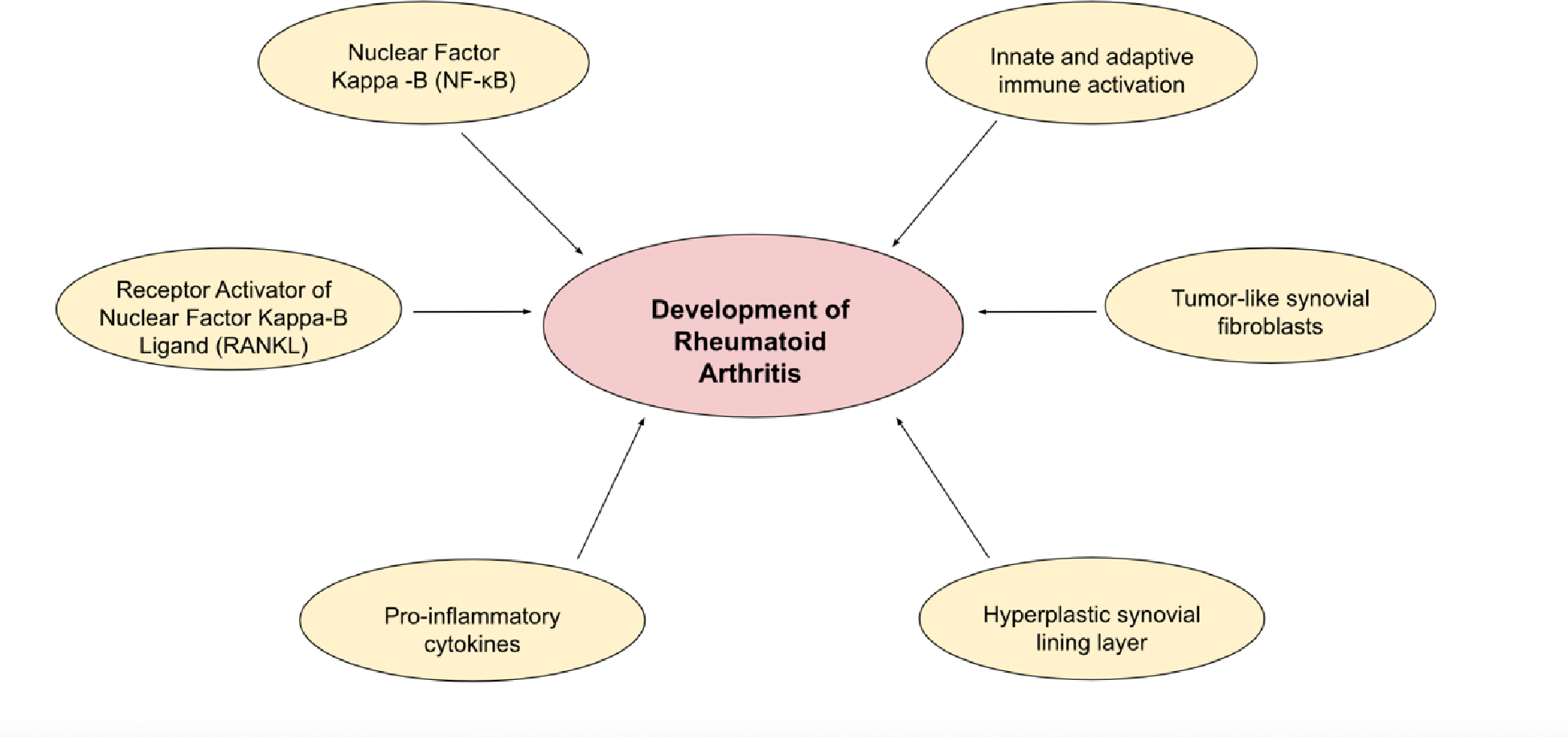
Inflammatory Pathways Contributing to the Pathophysiology of Rheumatoid Arthritis. This figure demonstrates the various mechanisms and inflammatory pathways contributing to the development of rheumatoid arthritis.

Each of these mechanisms illustrates the complex interplay of immune cells, cytokines, and autoantibodies in driving inflammation and bone erosion in RA (Figure 2). This aggressive remodeling and immune cell interaction underpin the chronic and self-sustaining nature disease progression as well as explains the many different therapies required to tackle different inflammatory processes. Important markers and inflammatory mediators such as IL-1β, IL-6, IL-7, IL-15, RANKL, and TNF-α are important when it comes to therapeutic measures, personalized medicine, and are key in understanding the complexities of RA.

### Methotrexate and Current Therapies in Rheumatoid Arthritis

#### (i) Further Insights into Methotrexate (MTX)

Treating RA patients with MTX can be challenging because there are varying degrees of response depending on the individual. To determine how patients may respond to MTX, one study evaluated 312 patients to assess clinical factors, such as age and levels of markers for inflammation. Their findings indicated that poorer response to MTX was associated with patients who had a younger age of disease onset, were rheumatoid factor positive, and had elevated levels of erythrocyte sedimentation rate [22]. Additionally, to improve the bioavailability of the drug, one study explored the use of microneedle patches for treatment, which is beneficial because it provides a localized response and reduces invasiveness. After treating RA guinea pigs with the microneedle patches, they found that there were reduced levels of key RA-associated diagnostic markers, less swelling and bone erosion, and minimal long-term side effects [23]. In all, MTX is a first-line therapy and cornerstone treatment for RA, but its effectiveness and bioavailability can differ based on the administration route. Similarly, treatment outcomes can vary due to influencing clinical factors. Given these limitations, MTX may not be sufficient as a monotherapy for all patients. Therefore, it is essential to explore additional therapies that may modify or enhance the effectiveness of MTX and assess how these treatments can impact the overall outcomes for RA patients.

#### (ii) Conventional Synthetic Disease Modifying Antirheumatic Drugs (DMARDs)

Conventional synthetic DMARDs include sulfasalazine, hydroxychloroquine, and leflunomide. Sulfasalazine is a pro-drug, metabolized into sulphapyridine and 5-aminosalicylic. It has an unclear mechanism but demonstrates anti-inflammatory and immunoregulatory properties [7,24]. Hydroxychloroquine is an antimalarial that possesses anti-inflammatory properties by suppression of autoreactive T cells [7,25]. Leflunomide works by arresting nucleotide synthesis, leading to inhibition of T lymphocyte proliferation and ultimately reducing immune reactions [26].

For the treatment of RA, there are many possible combinations when using conventional synthetic DMARDs. One common therapy is a triple combination of MTX, sulfasalazine, and hydroxychloroquine, while another possibility is a double therapy of MTX and leflunomide. In a study comparing the rates at which patients adhered to therapy, they concluded that while there were longer retention rates of therapy use with the triple combination compared to the double, the findings were not statistically significant [27].

When evaluating how MTX may interact with other DMARDs, one study looked at the incidence rate of serious adverse events with the combination of MTX + leflunomide versus MTX + sulfasalazine. They found that there was a better safety profile with MTX + leflunomide compared to MTX + sulfasalazine [28]. Yet, when directly comparing the toxicity and efficacy differences in monotherapy of leflunomide to MTX + leflunomide, there was no statistically significant difference [26]. Another study also revealed that there was no difference between the safety profiles of MTX + leflunomide and MTX+ hydroxychloroquine in RA patients who had inadequate response to MTX alone; however, the median time to remission was quicker with MTX + hydroxychloroquine, taking 11 months, compared to 16 months with MTX + leflunomide [29].

To further evaluate the adverse effects of these therapies, especially in RA patients with increased cardiovascular (CV) risk, one study compared hydroxychloroquine and MTX. In patients without pre-existing CV risk, both medications had similar rates of sudden cardiac arrest, ventricular arrhythmia, and major adverse cardiovascular events [30]. However, in patients with a history of heart failure who started hydroxychloroquine, there was an increased risk of CV-related adverse events. Additionally, regardless of prior heart failure history, initiating hydroxychloroquine was linked to a higher risk of hospitalization for heart failure [30].

Conventional synthetic DMARDs can be used in combination with MTX to reduce inflammation. However, when determining suitable treatment for a patient, these studies showed it is crucial to account for factors such as medication adherence, safety profile, duration of use, and the patient’s past medical history.

#### (iii) Biologic and Targeted DMARDs

Biologic DMARDs (bDMARDs) are another available therapy for RA patients. BDMARDs, infliximab and adalimumab, have been shown to have enhanced efficacy when used with MTX [31]. In addition, the combination of bDMARD + MTX led to a 24% decreased risk of RA patients having cardiovascular disease events [31].

Another study compared the monotherapy of bDMARD etanercept, a TNF inhibitor, to the monotherapy of MTX to assess whether it was more effective in maintaining remission rates. 49.5% of patients in the etanercept group-maintained remission compared to 28.7% of patients in the MTX group (p-value = 0.006) [32].

Likewise, tocilizumab, an IL-6 antagonist, is a bDMARD that can be used to treat RA as monotherapy or with MTX. IL-6 is a cytokine that can inhibit T-reg cells and impair the body’s ability to differentiate between self and non-self while also promoting inflammation [33,34]. Antagonizing IL-6 can be beneficial for treating RA patients who have not responded to previous standard treatments [34]. One study found that the combination of tocilizumab + MTX was more impactful in preventing the worsening of radiographic progression of joint damage compared to tocilizumab monotherapy [35]. However, there was no difference noted in the effectiveness of treating patients with early RA who had more joint damage and those with prolonged durations of RA [24].

Rituximab is also used for treating RA when standard therapeutics, such as MTX, are not adequate for controlling the disease [36]. This drug is an anti-CD20 monoclonal antibody that promotes B cell depletion by inducing cell death, and thus, modulating the immune system [37,38]. When comparing RA patients who maintained the standard dose versus switching to a low-dose regimen of rituximab once they demonstrated improvement in their condition, the low-dose regimen led to less chance of relapse and serious adverse events [36].

Another class of drugs that can treat RA are targeted DMARDs, such as JAK inhibitors tofacitinib and baricitinib. Their mechanism of action is to block the signaling of cytokines that promote inflammation [39]. These drugs can be used as monotherapy or with MTX. A study showed that there was remarked improvement in RA patients who were treated with JAK inhibitors after failure with MTX and other bDMARDs [39]. When directly comparing the safety profile and effectiveness of baricitinib and tofacitinib used in combination with MTX, they were similar. However, when it came to pain relief, baricitinib was more effective [40]. Additionally, another study showed that monotherapy baricitinib, when compared to MTX, showed better rates of remission of RA—62% and 48%, respectively (p-value = 0.03) [41]. Patients using baricitinib were also able to achieve remission quicker and had less radiographic progression in seropositive RA patients [41].

Finally, when comparing the first-line therapies of etanercept, adalimumab, and JAK inhibitors in RA patients, one study analyzed patient data from the CorEvitas RA Registry and demonstrated that there were no significant differences in the effectiveness of monotherapy or combination therapy with any of these treatments [42].

Although MTX is the most used first-line therapy for RA, bDMARDs and targeted DMARDs can be used concomitantly and can be effective in those who had an inadequate response to MTX. In some cases, bDMARDs and targeted DMARDs also may have a better efficacy and safety profile than MTX. These studies highlight the complexity of RA as a disease and importance of providing individualized treatment to achieve disease remission.

#### (iv) Non-steroidal anti-inflammatory drugs

Non-steroidal anti-inflammatory drugs (NSAIDs), such as ibuprofen and naproxen, can be used to reduce acute inflammation in RA by the inhibition of cyclooxygenase (COX) [43]. Non-selective NSAIDs can cause adverse side effects, such as gastrointestinal ulceration and renal injury [43,44]. Therefore, the administration of COX-2 selective inhibitors, like celecoxib, can mitigate some of these effects [43]. As NSAIDs are used intermittently to help with RA flare-ups, it is important to understand their interaction with standard medications, such as MTX. One study noted that low-dose MTX (less than 30 mg) was primarily safe to use alongside NSAIDs as it did not pose a significant risk of interacting or diminishing effectiveness [45]. Yet, when MTX is used alongside aspirin, the combination can increase toxicity levels in the liver and kidney dysfunction [45,46].

Altogether, these studies reinforce the value of having a multi-pronged approach for treating RA patients. While NSAIDS may not be the primary treatment for RA, they can be used to alleviate acute episodes of inflammation and provide therapeutic support alongside MTX.

#### (v) Glucocorticoids

Glucocorticoids (GCs) are used intermittently for acute flare-ups of RA, as they possess anti-inflammatory properties but are limited in use because of causing systemic toxicity, like increasing risk of osteoporosis and hyperglycemia [47,48]. Although GCs are used for short-term treatment in RA, the CareRA trial demonstrated their long-term benefits when used in the initial treatment with MTX of early RA patients, who had a favorable prognosis. This subpopulation had lower long-term utilization of NSAIDs and other analgesics compared to patients treated with MTX alone [49].

Short-term GC use can be beneficial for controlling sudden exacerbations of inflammation and reduce the future use of additional therapeutic support when used with MTX. Again, these findings emphasize the need for providing tailored treatments to maximize the therapeutic efficacy while minimizing risks.

Single Nucleotide Polymorphisms (SNPs) Impacting MTX Metabolism in RA Patients

When evaluating clinical studies published within the past five years exploring the impact of SNPs on MTX metabolism in RA patients, certain SNPs were associated with treatment failure and increased toxicity of the drug or its metabolite, MTX-7-OH, while others were correlated with increased tolerance or showed no significant correlation (Table 2).

When assessing the plasma of RA patients, one study concluded that the SNP rs4149081 (SLCO1B1 gene) was associated with higher concentrations of MTX and MTX-7-OH (p < 0.05) [50]. In the same study, SNP rs1476413 (MTHFR gene) TT or CT alleles were correlated with increased levels of MTX metabolites and severity of disease symptoms (p < 0.001) [50]. Similarly, another study identified the correlation of SNP rs1476413 (MTHFR gene) with MTX monotherapy failure after one to two years of treatment, alongside SNPs rs246240 (ABCC1 gene), rs2259571 (AIF-1 gene), and rs2274808 (SLC19A1 gene) [51]. Other SNPs that were associated with inadequate response to MTX include rs3821353 (ATIC gene) and rs7279445 (SLC19A1 gene) [52]. By contrast, the SNP rs10072026 (DHFR) was correlated with adequate response to MTX [52]. Likewise, a separate study identified SNPs with contrasting effects on MTX tolerance, with GGH-T401C (rs11545078) associated with increased intolerance and ABCC2-C24T (rs717620) with increased tolerance [53]. In another study, they found that out of the 20 patients who were unresponsive to MTX, 19 patients had SNP C3435T (ABCB1 gene) (p <0.001) [54].

SNPs may also play a role in influencing adverse drug effects. A study identified SNPs specifically on the SLCO1A2 gene of RA patients treated with MTX monotherapy for a minimum of six months. The G550A polymorphism was correlated with increased risk of adverse drug effects with MTX [55]. Another study identified that increased adverse effects were associated with the SNP 1994A > G (FPGS gene) in RA patients who had been treated with MTX for 24 weeks [56].

Finally, genes such as MTHFR, which have SNPs known to influence MTX metabolism, may also contain variants that do not impact metabolism. For instance, one study evaluating SNPs in early RA patients on MTX monotherapy found that C677T (rs1801133) and A1298C (rs180113) on the MTHFR gene were not associated with changes in MTX metabolism [57].

Overall, these findings highlight the role that SNPs have in influencing MTX rates of metabolism, treatment efficacy, and adverse effects. It also underscores the complexity and the potential that the identification of genetic markers, like SNPs, can play when determining the best individualized treatment for RA patients.

### SNPs Impacting MTX Metabolism in Different Racial, Ethnic, and Population-Based RA Patient Groups

Identifying SNPs that influence MTX metabolism in various racial, ethnic, and population-based groups is crucial, as genetic differences can provide valuable insights into how patients respond to therapy.

Regarding race-specific research related to SNP and MTX metabolism, one study found that ABCC2 IVS 23+56 T → C SNP was associated with MTX toxicity-related dose adjustments or drug discontinuation in Caucasians with RA but not in African Americans [58]. This intronic SNP may serve as a genetic marker of MTX toxicity or occur in linkage disequilibrium with a variant that determines toxicity in Caucasian populations. Likewise, another study that examined genetic and population-based differences in MTX efficacy, toxicity, and related allelic variations among RA patients of African American and Caucasian populations that the 1298A allele in Caucasians was associated with a significant increase in MTX-related adverse events, whereas in African Americans, the rs4846051C allele and its haplotypes were associated with MTX toxicity [59]. Yet, other factors that may play a role in MTX efficacy and metabolism are those of lifestyle, such as differences in folate status [60,61], socioeconomic factors, diet, lifestyle, and education [62]. These factors likely contribute to population-specific variability in MTX adverse events, independent of SNPs or genetics [62]. For example, a survey on RA patients found that African American patients exhibited higher risk aversion related to MTX toxicity (adjusted OR 8.4, 95% CI 3.1–23.1), emphasizing risks over benefits compared to white patients [63]. Additionally, studies often lack large and representative African American sample populations, limiting their ability to draw definitive conclusions [62]. Enhanced risk communication, aversion, and education as well as emphasizing the expected benefits could reduce disparities in treatment acceptance, and eventually, patient outcomes.

Other studies have focused on findings related to the impact of SNPs on MTX metabolism within the context of ethnicity and population-based groups. In contrast to the association of ABCC2 IVS 23+56 T → C SNP in Caucasians previously mentioned, this SNP had higher allele frequencies in Japanese populations [64]. In another study conducted in Malaysia on 647 RA patients, none of the SNPs were significantly associated with MTX efficacy or toxicity based on the Chi-square test and logistic regression [65]. However, stratification by ancestry revealed that among patients of Malay descent, two SNPs—ATIC C347G (rs2372536) (OR 0.5478, 95% CI 0.3396–0.8835, p = 0.01321) and ATIC T675C (rs4673993) (OR 0.5247, 95% CI 0.3248–0.8478, p = 0.008111)—were significantly associated with an adequate MTX response (p <0.05), but no SNPs were linked to MTX toxicity [65]. A study conducted on Iraqi patients in 2024 found that gene polymorphisms of the TYMS gene: TCrs2853741 and ACrs260641, predict non-responsiveness while the CTAT genotype predicts a successful MTX response [66]. The same authors later concluded in a separate study of Iraqi patients that TTrs1801131 genotypes were associated with unresponsiveness, and the G-carried allele for rs1801131 has an association with response to MTX [67]. Looking at Egyptian adult RA patients treated with MTX, a study identified MTHFR A1298 CC as the primary SNP likely to predict both MTX toxicity and efficacy [68]. Finally, a 2017 meta-analysis on European RA patients concluded the significant association between the toxicity of MTX and the RFC-1 80G > A (rs1051266) polymorphism [69]. These various studies demonstrate that there can be significant associations between SNP and MTX metabolism in different ethnic and population-based groups.

Despite some insights, there remains no clear consensus on how MTX metabolism varies across many racial, ethnic, and population-based groups and how SNPs may contribute, as most past research focuses primarily on comparisons specifically between African American and Caucasian RA patients. Knowing these genetic differences allows for tailored therapy, enabling patients to respond more effectively to treatment and minimizing adverse outcomes [70]. To bridge existing gaps, future studies must prioritize larger, diverse cohorts and focus on integrating genetic, environmental, and socioeconomic variables into treatment strategies, ensuring equitable care for all patients with RA.

## Conclusion

RA is a complex, systemic autoimmune disease that utilizes MTX, a DMARD, as the first-line treatment. However, its efficacy can vary significantly between patients due to multiple influences. Understanding how MTX interacts with other medications commonly used in treatment with RA can provide insight into personalizing therapies to optimize clinical outcomes. Similarly, SNPs can impact the response to MTX treatment by affecting its drug metabolism and may be associated with adverse drug effects. Due to the presence of SNPs, tailoring treatment to individuals based on their genetic profile, including racial, ethnic, and population-based background, is valuable in providing personalized care for the management of RA. Future research into pharmacogenomics and identification of genetic markers associated with MTX metabolism can further our understanding of RA pathogenicity. Advancements in our knowledge of genetic polymorphisms on drug efficacy can enhance the effectiveness of RA treatment and, therefore, improve therapeutic outcomes.

## Figures and Tables

**Table 1:** Mechanism of Actions of Drugs in the Treatment of Rheumatoid Arthritis. This table highlights many of the therapeutics discussed for RA treatment, focusing specifically on an overview of the relevant mechanism of action.

Drug	Mechanism of Action
methotrexate	Affects the folate metabolism pathway via inhibition of dihydrofolate reductase to disrupt DNA synthesis and purine metabolism
sulfasalazine	Unclear mechanism of action; pro-drug metabolized into sulfapyridine and 5-aminosalicylic
hydroxychloroquine	Suppression of autoreactive T cells
leflunomide	Inhibition of T lymphocyte proliferation via arresting nucleotide synthesis
etanercept	TNF inhibition
tocilizumab	IL-6 antagonist
rituximab	Anti-CD20 monoclonal antibody that induces cell death to to promote B cell depletion
tofacitinib/baricitinib	Inhibition of janus kinases (JAK) to block signaling of cytokines that induce inflammation
non-steroid anti-inflammatory drugs (NSAIDs)	Reduction of acute inflammation via inhibition of cyclooxygenase (COX)

**Table 2:** Impact of Single Nucleotide Polymorphisms (SNPs) on MTX Metabolism. This table summarizes the SNPs and their relationship with MTX metabolism.

SNPs	Impact of MTX Metabolism
rs4149081 (*SLCO1B1* gene)	Associated with higher concentrations of MTX and MTX-7-OH
rs1476413 (*MTHFR* gene)	Associated with increased MTX metabolite levels and severity of disease symptoms and with MTX monotherapy failure post-treatment for one to two years
rs246240 (*ABCC1* gene)	Associated with MTX monotherapy failure post-treatment for one to two years
rs2259571 (*AIF-1* gene)	Associated with MTX monotherapy failure post-treatment for one to two years
rs2274808 (*SLC19A1* gene)	Associated with MTX monotherapy failure post-treatment for one to two years
rs3821353 (*ATIC* gene)	Inadequate treatment response to MTX
rs7279445 (*SLC19A1* gene)	Inadequate treatment response to MTX
s10072026 (*DHFR gene)*	Adequate treatment response to MTX
GGH-T401C/ rs11545078	Increased intolerance to MTX treatment
ABCC2-C24T/ rs717620	Increased tolerance to MTX treatment
C3435T (*ABCB1* gene)	Unresponsive to MTX treatment
G550A (*SLCO1A2* gene)	Increased risk of MTX adverse drug effects
1994A > G (*FPGS* gene)	Increased risk of MTX adverse drug effects
C677T/ rs1801133 (*MTHFR* gene)	Not associated with impacting MTX metabolism
A1298C/rs180113 (*MTHFR* gene)	Not associated with impacting MTX metabolism

## Key points, outstanding questions, and challenges

- Methotrexate (MTX) metabolism in rheumatoid arthritis (RA) patients is influenced by combination therapies and genetic polymorphisms (SNPs), impacting treatment efficacy and toxicity.
- Further research is essential to better understand the complex interplay of drug interactions, genetic factors (SNPs), and demographic variables (race, ethnicity, socioeconomic factors) in MTX metabolism to improve therapeutic outcomes for all RA patients.
- Are there specific biomarkers (beyond genetic markers) that can predict how a patient’s MTX metabolism will be affected by combination therapies, allowing for more proactive and personalized treatment decisions?
- How do gene-environment interactions (e.g., the interplay between specific SNPs and factors like diet, smoking, or folate levels) influence MTX metabolism and treatment outcomes in RA patients?
- Can population-specific pharmacokinetic and pharmacodynamic studies of MTX be conducted to establish more tailored dosing guidelines for different ethnic groups?
- Currently, there is not adequate literature to cover neither the epigenetics of many ethnic groups nor the socioeconomic factors involved in RA. Additional studies are warranted in these areas.
